# Digital biomarker applications across the spectrum of opioid use disorder

**DOI:** 10.1080/28324765.2023.2240375

**Published:** 2023-08-01

**Authors:** Marc Rigatti, Brittany Chapman, Peter R Chai, David Smelson, Kavita Babu, Stephanie Carreiro

**Affiliations:** a Department of Emergency Medicine, UMass Chan Medical School, Worcester, MA, USA; b Department of Emergency Medicine, Harvard Medical School, Boston, MA, USA; c Department of Psychiatry, UMass Chan Medical School, Worcester, MA, USA

**Keywords:** opioid use disorder, addiction, digital biomarker, wearable sensors, opioid withdrawal

## Abstract

Opioid use disorder (OUD) is one of the most pressing public health problems of the past decade, with over eighty thousand overdose-related deaths in 2021 alone. Digital technologies to measure and respond to disease states encompass both on- and off-body sensors. Such devices can be used to detect and monitor end-user physiologic or behavioral measurements (i.e. digital biomarkers) that correlate with events of interest, health or pathology. Recent work has demonstrated the potential of digital biomarkers to be used as a tools in the prevention, risk mitigation and treatment of OUD. Multiple physiologic adaptations occur over the course of opioid use and represent potential targets for digital biomarker-based monitoring strategies. This review explores the current evidence (and potential) for digital biomarkers monitoring across the spectrum of opioid use. Technologies to detect opioid administration, withdrawal, hyperalgesia and overdose will be reviewed. Driven by empirically-derived algorithms, these technologies have important implications for supporting the safe prescribing of opioids, reducing harm in active opioid users and supporting those in recovery from OUD.

## Introduction

Opioid use disorder (OUD) is one of the most pressing public health problems of the last decade (Volkow & Collins, 2017). Both in the United States (US) and globally, the incidence and prevalence of OUD have escalated at alarming rates. In 2019, an estimated 1.6 million people in the US met the criteria for OUD (SAMHSA, 2020) and are thus at risk for contributing to the staggering opioid overdose death statistics. In the same year, 71% of all drug overdose deaths in the US (49,860 of 70,630) were attributable to an opioid (Mattson et al., 2021). This equates to roughly five lives lost every hour. The current odds of dying from an opioid overdose exceed those of dying from a motor vehicle crash or firearm injury (Mattson et al., 2021). The scale and scope of the epidemic have compelled federal authorities such as the US Department of Health and Human Services and the Surgeon General to declare combating opioid misuse as one of the highest public health priorities facing our nation today (Substance A, Mental HSAUS, the Surgeon G, Others, 2018).

OUD can occur after exposure to opioids via *any* route; this includes both illicit and prescription opioid use. Over the last 10 to 15 years, prescribing opioids has increasingly been recognized as a high-risk event. The steep upwards trajectory in opioid prescribing rates from the late 1990s to the early 2010s was highly correlated with an increase in prescription drug misuse, OUD, initiation of IV drug use and overdose-related death (Kolodny et al., 2015; Muhuri et al., 2013). Not surprisingly, many people with OUD report their first exposure to opioids that was a prescription opioid: either through their own legitimate prescription from a healthcare provider or access to opioids that were prescribed to someone else. The percentage of individuals in treatment for heroin use disorder that reports their initiation of opioid use to be via prescription opioids rose from 20% in the 1960s to 75% in 2000s (Cicero et al., 2014). Furthermore, the misuse of opioid analgesics has been described as a strong predictor of opioid-related death. Not every person who receives a prescription for an opioid misuses the drug or progresses to OUD; however at present, there is no way of identifying which patients are at the highest risk to do so. As a result, the current mantra is restrict access/exposure to opioids for as long as possible; in other words, “keep opioid-naive patients opioid naive” (Babu et al., 2019; Delgado et al., 2018; Strayer et al., 2017). Unfortunately, opioids are the current standard of care for treatment of severe acute pain, putting healthcare providers in the difficult position of needing to carefully balance the desire to alleviate suffering with the risk of initiating the pathway to OUD (Strayer et al., 2017).

Opioids cause predictable physiology in therapeutic and toxic doses. Repeated exposure to opioids even at therapeutic doses results in multiple physiologic adaptations: these include tolerance, hyperalgesia, dependence and withdrawal. These physiologic adaptations drive behavior surrounding opioid use patterns and have a role in the development (and diagnosis) of OUD. In fact, several of them (including tolerance and dependence/withdrawal) are included in the Diagnostic and Statistical Manual of Mental Disorders (DSM V) criteria for OUD (Hasin et al., 2013). Non-invasive sensors have the potential to provide continuous, passive and real-time physiologic monitoring, which in the setting of opioid use could identify both acute adverse events (e.g. overdose) and dynamic changes in response to the drug (e.g.tolerance or withdrawal). Digital devices (including fitness trackers, smart phones and other on- and off-body sensors) can thus be used to detect and monitor end-user physiologic measurements (i.*e. digital biomarkers*) that correlate with events of interest, health or pathology (Bent et al., 2020; Montag et al., 2021; Wright et al., 2017) (in this case, response to opioids). Despite recent advances in other areas of medicine and even in other substance use disorders (SUDs) (Akinosun et al., 2021; Chatterjee et al., 2020; Davis-Martin et al., 2021; Gullapalli et al., 2019; Natarajan et al., 2013; Saxena et al., 2021; Su et al., 2021), there is a paucity of clinically available digital biomarker-based tools related to opioid use; however, many are in the research and development pipeline and show great potential.

The importance of digital health tools in the diagnosis, prevention and treatment of OUD has garnered widespread attention in recent years, with a rapidly growing body of literature and expert opinions. Prior reviews have demonstrated their potential (and growth) in SUD space. In 2020, Goldfine et al. provided a review on wearables and wireless technologies for SUDs: in the context of OUD, they noted several early-stage technologies, including wearable and ingestible sensors with potential applications in OUD (Goldfine et al., 2020). In 2020, Nuamah et al. reviewed mobile applications for OUD management and found that despite a steady rise in the availability of (largely clinician facing) apps over the preceding 10-year period, they did not find any studies to test the efficiency of such apps in OUD (Nuamah et al., 2020). In 2021, Beaulieu et al. conducted a review of the grey literature on artificial intelligence (AI)-based interventions for OUDs. They identified 29 unique interventions in the following categories: smartphone applications, healthcare data-related interventions, biosensor-related interventions and digital/virtual interventions (Beaulieu et al., 2021). Oesterle et al. reviewed available wearable remote-monitoring solutions for nonalcohol, nontobacco SUD (including OUD), and found that the majority of studies focused on the detection of intoxication (Oesterle et al., 2022). Gahdia et al. recently reviewed available AI applications for OUD and noted the majority focused on risk prediction or surveillance and monitoring using EHR, survey and/or social media data (Gadhia et al., 2022). Prior work has either included OUD under the larger blanket of SUD and/or focused on various aspects of OUD where digital tools can be used, including prescribing patterns, clinician education and psychosocial interventions. Prior work has not, to our knowledge, focused specifically to review digital health tools for detection of opioid physiology across the spectrum of OUD. Opioid physiology is objective, dynamic (i.e. changes over time with repeated use) and less influenced by behavioral variations that other phenomenon (e.g. provider prescribing practices), making it an excellent monitoring target and a lens through which to evaluate digital health tools.

The current review explores digital biomarkers applicable to therapeutic opioid use and OUD, using the distinct physiologic effects of and adaptations to opioids as key junctures to categorize these applications. Specifically, it will explore applications for the detection of opioid use/adherence, withdrawal/dependence, tolerance/hyperalgesia and overdose (Table 1). Additionally, we will discuss potential future applications of these systems to mitigate the development of OUD among those who are at high risk, support harm reduction strategies and provide support to those with OUD seeking treatment and recovery.

**Table 1.** Overview of mHealth technologies for various domains of opioid effect

Opioid Effect	Definition	Example of mHealth Technology to Monitor
Opioid Administration	Activation of the opioid receptor by and opioid, and the resultant physiologic response	Digital pill system (Chai, Carreiro, Innes, Rosen, et al., 2017) (Chai, Carreiro, Innes, Chapman, et al., 2017)
		Wearable sensors (Carreiro et al., 2016) (Kennedy et al., 2015) (Mahmud et al., 2018) (Gullapalli et al., 2021) (Salgado García et al., 2022)
Adherence	Taking opioids as prescribed	Wearable sensors (Carreiro et al., 2016) (Mahmud et al., 2018) (Gullapalli et al., 2021)
Withdrawal	Syndrome characterized by dysphoria, anxiety, diffuse myalgias, abdominal pain, vomiting, diarrhea and insomnia	Wearable sensors (Chintha et al., 2018) (Kulman, Venkatasubramanian, et al., 2021) (Lambert et al., 2022)
Dependence	Continued opioid exposure is required to prevent withdrawal symptoms	
Tolerance	Requirement of escalated doses to achieve the same effect	Wearable sensors
Opioid Induced Hyperalgesia	Paradoxical increase in sensitivity to pain associated with opioid therapy	
Overdose	Toxic effect of opioids which includes central nervous system depression and respiratory depression	Smartphone-based detection system (Nandakumar et al., 2019) Wearable detection system Roth et al., 2021
		Implantable naloxone delivery system (Dhowan et al., 2019) (Imtiaz et al., 2021) (Chan et al., 2021)

## Recognition of opioid administration

An area ripe for digital health solutions is therapeutic opioid monitoring. Opioids are typically prescribed to be taken on as as-needed basis. This puts some of the decisions about dosing on the patient and/or their caregiver including how often and how long to take opioids for (McCarthy et al., 2021). As previously discussed, the therapeutic opioid use can be an inciting factor in the development of OUD. At the same time, changes in opioid use patterns can suggest developing tolerance or dependence but may also suggest worsening of underlying disease that may require medical intervention. Understanding the context in which people use opioids can lend important insights. Traditional methods to measure opioid use include self-report and biologic specimen (blood, urine and saliva) assays; both have significant shortcomings. Inconsistent self-report and test interpretation limit applicability in the acute setting of opioid treatment (Heit & Gourlay, 2004; N. Katz & Fanciullo, 2002; Moreno et al., 2019). The former is subject to recall bias and intentional under-reporting to avoid stigma or negative consequences. The latter is subject to limited windows of detection and lack of granular information about timeline of use.

One effort to generate digital biomarkers of therapeutic opioid use includes the use of digital pill systems. In these connected systems, radio-frequency identification (RFID) tags are embedded into gelatin capsules in which pharmaceuticals of interest (e.g. the oral opioid oxycodone) can be packaged. Once ingested, the sensors are activated by chloride ions in the stomach and emit a signal to an externally worn reader. This reader has multiple form factors including a lanyard-based system, cutaneous patch, wristband or pendant. The system can then send objective information regarding the exact patterns and timing of doses to the patient’s healthcare provider. To date, two digital pill systems (Proteus Digital Health and eTectRx) have obtained US Food and Drug Administration 510K clearance as medication ingestion event monitors. Digital pills containing opioid pharmaceuticals have been shown to be both accurate in report opioid ingestion and acceptable to end users (Chai, Carreiro, Innes, Chapman, et al., 2017; Chai, Carreiro, Innes, Rosen, et al., 2017).

A second approach to opioid use detection utilizes wearable sensors, which can measure a number of physiologic parameters, including heart rate and rhythm, accelerometry, skin temperature, respiratory rate and blood oxygen saturation. Opioid administration induces reliable changes in the central nervous, cardiovascular and respiratory systems, making continuous physiologic monitoring and attractive strategy for opioid detection. Initial early studies focused on the feasibility of wearable sensors to detect opioid use, that is, to determine whether there was a detectable signal in measurable physiology after opioid administration. Kennedy et al. enrolled 40 individuals with polysubstance use disorder and collected 50 heroin-only use events from a field environment, and noted a significant decrease in heart rate immediately prior to heroin consumption using a chest worn sensor (AutoSense) (Ertin et al., 2011; Kennedy et al., 2015). Other investigators enrolled emergency department (ED) patients (*N* = 30) to wear the Affectiva Q Sensor (Affectiva, Boston, MA) while receiving a single dose of intravenous opioids. Opioid administration produced a distinct biometric profile (i.e. increased skin temperature and decreased motion in the X axis) that correlated with opioid injection (Carreiro et al., 2016). Using various machine learning models (including K-Nearest Neighbors, decision trees and extreme gradient boosting approaches), opioid use events were identifiable from these data with high accuracy (Mahmud et al., 2018). In a follow-up study, the same investigators enrolled hospitalized patients and observed repeated opioid administrations using the Empatica E4 wrist sensor (Empatica, Milan, Italy) (Gullapalli et al., 2021). Using sensor data from 339 opioid administrations, they developed a machine learning model to detect opioid administration within 9 min of administration with 77% specificity and 80% sensitivity. Similar results have been demonstrated with out-of-hospital opioid administration: Salgado-Garcia et al. monitored 46 adults taking oral opioids after dental surgery using the Empatica E4 and developed a machine learning model to detect opioid use with sensor data with 81% sensitivity and 88% specificity (Salgado García et al., 2022).

Currently, healthcare providers and patients lack objective tools to monitor the development of problematic behaviors *before* they lead to OUD and/or to predict the transition to OUD. Detection of opioid administration and adherence has multiple potential clinical applications in this space by providing passive and objective data during therapeutic opioid use. Ingestible or wearable sensors can be used to monitor patterns of opioid use, signal excessive use, and possibly identify early stages of OUD, or pre-addiction (McLellan et al., 2022). These data could be leveraged for both patient and provider use. For patients, opioid use patterns could be used to trigger educational content, encourage alternative pain control strategies, and/or provide motivational messaging, empowering them to proactively manage opioid use. Preliminary studies have already demonstrated acceptability of wearable sensor monitoring of opioids in patients in this setting. In a qualitative study of perceptions on wearables for therapeutic opioid monitoring, Chapman et al. found that participants reported increased awareness surrounding opioid use, increased accountability, and a sense that providers cared more about their wellbeing associated with sensor use (Chapman et al., 2022). For providers, digital biomarkers data could be harmonized with electronic health record (EHR) and/or patient reported outcomes data to assess risk for transition to OUD, identify maladaptive use patterns, and determine need for adjunctive analgesics strategies.

## Opioid withdrawal and dependence detection

Opioid dependence and withdrawal are related physiologic adaptations to opioid use. Repeated exposure to opioids leads to opioid *dependence*, where continued exposure is required to prevent *withdrawal* symptoms (Pergolizzi et al., 2020). Opioid withdrawal is a syndrome characterized by dysphoria, anxiety, diffuse myalgias, abdominal pain, vomiting, diarrhea and insomnia. Opioid-dependent individuals generally describe withdrawal as profoundly aversive, and will go to great lengths to avoid it (Lai et al., 2021). In fact, individuals with OUD will often describe a change in motivation to use opioids over time, initially citing a desire to achieve euphoria, and eventually using opioids simply to avoid the pain of withdrawal (Pergolizzi et al., 2020). Opioid dependence and withdrawal are thought to be driven by a number of neurobiologic changes that result in overall neuronal hyperactivity; these include compensatory changes downstream from opioid receptors on the cellular level, and alteration in key neuroactive compounds (i.e. overall increase in glutamate and norepinephrine activity and overall decrease in dopamine and serotonin activity) (Burma et al., 2017; Colvin et al., 2019).

The distinct and often dramatic physical manifestations of opioid withdrawal make it an attractive target for digital biomarker monitoring; however, there is a paucity of literature exploring this option. One study used wearables to observe precipitated withdrawal and subsequent recurrent opioid toxicity, which commonly follow the administration of the mu-opioid receptor antagonist naloxone (Kanof et al., 1992; Rzasa Lynn & Galinkin, 2018). Using the Empatica E4 wearable wrist sensor and a signal-processing approach to sensor data analysis, investigators observed ED patients who presented immediately after an opioid overdose treated with naloxone (Chintha et al., 2018). A distinct shift in physiology was noted approximately 90 min post-naloxone administration, namely, increase in skin temperature, decrease in HR and decrease in overall movement parameters. With regard to accelerometry data, and there were again qualitative changes in biometric pattern noted. In the period where naloxone was still active (within 90 min of naloxone administration), participants showed high levels of short amplitude (or fidgeting type) movements, consistent with opioid withdrawal. After naloxone wore off (approximately 90 min after administration), the distribution of amplitudes leveled off, signaling a decrease in the short amplitude movements. In a follow-up study in the same cohort, the authors took a different approach and used machine learning to identify specific periods of withdrawal in the sensor data (which correlated with Clinical Opiate Withdrawal or COWS scores). The best performing model was a Random Forest classifier with an area under the curve (AUC) for the receiver operating characteristic (ROC) curve of 0.9997 (Kulman, Chapman, et al., 2021). Opioid withdrawal was again associated with increased EDA and decreased heart rate/heart rate variability. Other investigators have used wearables worn on the trunk to assess withdrawal. Lambert et al. evaluated 23 adults undergoing opioid withdrawal in a research lab setting using a wearable accelerometer on the sternum (Lambert et al., 2022). Oscillatory patterns in accelerometry data demonstrated statistically significant correlation with both COWS scores and change in COWS score over time. Although not described in the literature to date, off-body sensor systems could also be leveraged to detect opioid withdrawal. For example, digital phenotyping from smartphone data could be used to identify features of withdrawal similar to its application to identify and grade anxiety symptoms (Jacobson & Feng, 2022; Jacobson et al., 2020).

Wearable sensor applications to opioid dependence have been studied even less than withdrawal, although previously mentioned studies on opioid administration do provide some insight. In addition to noting biometric changes with opioid administration, Carreiro et al. noted qualitative differences in these changes between participants maintained on chronic opioids compared to those produced by opioid-naive participants particularly in movement parameters (Carreiro et al., 2016). In this study, ED patients were monitored continuously with a wrist-mounted sensor (Affectiva Q Sensor) while they received a single dose of a therapeutic opioid intravenously. The sensor measured tri-axial accelerometry, electrodermal activity and skin temperature. Overall, the participants with chronic opioid use showed a relative decrease in short amplitude movements (i.e. fidgeting-type movements) on the x-axis compared to opioid-naive participants. This suggests individuals who use opioids chronically may have a unique digital biomarker prior to opioid administration that resembles opioid withdrawal (based on similarities to precipitated withdrawal resolution in opioid overdose patients (Chintha et al., 2018), and is consistent with expected behavior/physiology in opioid dependence.

For individuals working toward recovery from OUD, specifically those in treatment with buprenorphine, objective monitoring of withdrawal coupled with detection of opioid administration (as described above) would offer more tailored treatment solutions. Quantification of withdrawal would be incredibly advantageous for the buprenorphine induction process (identifying readiness, quantifying response and guiding dosing), and is a potential target for a future digital therapeutic. And once treatment is underway, deploying a continuous detection system may help reinforce buprenorphine adherence. More importantly, accurate and real-time notifications of buprenorphine adherence patterns or withdrawal symptoms can be coupled to just-in-time adaptive (JITA) interventions tailored to individual needs, an approach that has been identified as promising for SUD treatment but lacking rigorous research (Carpenter et al., 2020; Marsch, 2020; Perski et al., 2021). Intervention possibilities include those in real time (i.e. positive reinforcement in the case of adherence, or motivational messaging/well-checks in the case of non-adherence or withdrawal symptoms) and retrospective data review to identify patterns and correlates (i.e. correlating sleep, exercise, geolocation and other contextual data).

## Opioid tolerance and hyperalgesia monitoring

Opioid tolerance and opioid-induced hyperalgesia (increased sensitivity and response to pain) are distinct but overlapping phenomena. Opioid tolerance refers to the process by which escalating doses are required to achieve the same effect and can refer to pharmacokinetic or pharmacodynamic tolerance (Dumas & Pollack, 2008; M. C. Lee et al., 2014). *Pharmacokinetic* tolerance, or the need for higher doses to achieve the same drug concentration, occurs due to drug-induced changes in absorption, distribution, metabolism and excretion. *Pharmacodynamic* tolerance refers to the need for higher doses to achieve the same clinical effects, both desirable therapeutic effects (i.e. analgesia) and adverse effects (i.e. sedation and respiratory depression). Opioid tolerance develops differentially across effects; for example, tolerance to analgesia occurs much more rapidly than tolerance to respiratory depression or constipation (Hayhurst & Durieux, 2016). This creates a dangerous scenario, whereas a “tolerant” patient can receive tremendous doses of opioids that do not provide effective analgesia but place them at high risk for respiratory depression. Opioid-induced hyperalgesia (OIH) is defined as a paradoxical increase in sensitivity to pain associated with opioid treatment (Marion Lee et al., 2011). OIH has been demonstrated in individuals maintained on methadone (Zahari et al., 2016) patients with chronic pain (Cohen et al., 2008) and even patients with short-term intra-operative opioid exposure (Hayhurst & Durieux, 2016).

At present, the most common methodology employed for detecting and measuring OIH is quantitative sensory testing (QST) (Edwards et al., 2011, 2016; Higgins et al., 2019; N. P. Katz et al., 2015; Wasserman et al., 2015). QST involves stimulating the patient with quantified sensory stimuli (e.g. vibration, temperature, pressure, electrical stimuli) and determining thresholds for sensory perception based on the patient’s response. This method has been used with varied degrees of success. It appears dependent on the specific method of QST and the presence of OUD or use of N-methyl-D-aspartate (NMDA) antagonists, particularly methadone (Higgins et al., 2019). Despite the quantitative nature of the applied noxious stimuli, QST has limitations. There are subjective components to the test that are dependent upon patient motivation and participation. Patients can intentionally worsen performance, resulting in substantial intra- and interindividual variations (Higgins et al., 2019).

Multiple groups have employed various other arrays of wearable biosensors, EEG, pupillary unrest under ambient light, facial expression analysis, fMRI and infrared optical spectrometry with the goal of quantifying pain using measurable physiologic data (Chu et al., 2017; Dundar et al., 2018; Eisenried et al., 2018; J. Lee et al., 2019; Loggia et al., 2011; Seitsonen et al., 2005; Susam et al., 2018; Treister et al., 2012). For example, Chu et al. used blood volume pulse (BVP), electrocardiogram (ECG) and skin conductance level (SCL) to quantify pain. Healthy subjects were exposed to electrical stimulation to assess self-reported pain response, and machine learning models were developed to predict pain intensity with a mean accuracy of 75% (Chu et al., 2017). Othan et al. used facial expressions, ECG, electromyogram (EMG), and EDA data to build machine learning models that predict pain (Othman et al., 2022). Recent work employing machine learning methods has demonstrated promising results that indicate objective measurement of pain is feasible (Ben-Israel et al., 2013; Chu et al., 2017; Gram et al., 2015; M. C. Lee et al., 2014; Lin et al., 2018; Othman et al., 2022; Susam et al., 2018). However to our knowledge, non-invasive wearable sensors have not been investigated for detection or measurement of OIH or tolerance. Such successes indicate that similar methods may be applied for detecting and measuring OIH.

Objective measures of OIH and tolerance would provide tremendous advantages over current standard-of-care strategies (patient reported outcomes). An intuitive response to escalation of patients’ pain is to increase opioid analgesics; however in the case of OIH, this will paradoxically worsen pain. The ability to identify the onset/presence of OIH would allow providers (and patients) to understand when opioids are causing more pain than they treat, and when alternative therapies are indicated. In the case of tolerance, digital biomarkers could provide a personalized opportunity to titrate opioid therapy, improving analgesia and decreasing risk of overdose. Numerous compounds are under investigation that may attenuate OIH (including NMDA antagonists such as ketamine (Joly et al., 2005; Laulin et al., 2002; Marion Lee et al., 2011) and tolerance (including methylnaltrexone and NMDA receptor antagonists (Adam et al., 2008; Corder et al., 2017; Roeckel et al., 2016); digital biomarkers could also serve as precision targets to limit these physiologic adaptations to opioid therapy.

## Opioid overdose detection

Other efforts in this space have focused on the detection and treatment of arguably the most concerning complication of OUD opioid overdose. Opioid overdose results in a characteristic constellation of physiologic changes including decreased respiratory rate, bradycardia (decreased heart rate), hypothermia (decreased core body temperature), hypoxia (low blood oxygen)/hypercapnia (elevated blood carbon dioxide) and decreased sympathetic activation. These findings can be characterized through the use of wearable, cutaneous or contactless devices.

In 2019, Nandakumar and colleagues described a mobile phone-based detection system for opioid overdose (Nandakumar et al., 2019). The investigators used the microphone and speaker from a standard mobile phone as a short range sonar system to detect chest wall movement indicative of respiratory effort in the target user. The resultant algorithm was able to detect respiratory depression with 87% sensitivity and 89% specificity, and opioid-induced apnea with 96% sensitivity and 98% specificity. Another group of investigators used the Spire Health Tag (Spire Health, 2022), which passively measures respiratory rate and physical movement, to monitor 16 individuals who opioids in a field setting (Roth et al., 2021). They were able to capture over 1600 h of data, suggesting feasibility of respiratory rate monitoring in this population: however despite five reported overdoses, their sensor data did not demonstrate acute respiratory depression. The authors concluded that more sensitive sensors and triangulation of multimodal data would likely be needed to accurately capture opioid overdoses.

Others have proposed mechanisms to not only automate detection but also treatment of an opioid overdose using wireless devices. Following a similar theme of real-time overdose management, Dhowan et al. proposed a prototype for a closed-loop delivery system which would automatically deliver a subcutaneous dose of the opioid antagonist naloxone to reverse overdose if respiratory depression was detected (Dhowan et al., 2019). The system was tested in animal models, and successful implantation, wireless activation, and release of drug was demonstrated. Imitiaz et al. proposed a similar wearable device, which includes a microcontroller, pulse oximeter, hypodermic needle and reservoir of the opioid antagonist nalmefene (Imtiaz et al., 2021). On detection of a pulse ox value below 90% hemoglobin oxygen saturation (SpO2), the device actives, inserts the sterile needle and delivers a therapeutic dose of nalmefene subcutaneously. Chan et al. developed and tested a device that is worn as a patch on the lower abdomen, uses accelerometry to monitor respiratory status, and then autoinjects naloxone upon detection of respiratory depression (Chan et al., 2021). The device was tested in both simulated apneic events in healthy lab participants and in a supervised opioid injection facility. Respiratory rate detection was comparable to a gold standard device, and naloxone was successfully injected upon detection of apnea (in simulated events).

Although overdose detection devices are promising, additional work is needed prior to clinical deployment. A standalone device that detects opioid overdose is only valuable if it is coupled to a mechanism for rapid intervention once detected. Several intervention types have been proposed ranging from an automated call to a support person or emergency medical services to automated delivery of antidotal therapy as described above. The automated antidote delivery devices are particularly attractive. Monitoring for opioid toxicity to identify the need for repeat (or initial) naloxone dosing would enhance safety of both in- and out-of-hospital opioid use. However, several critical barriers must be addressed including robust protection against unnecessary dosing. And although preliminary studies have shown 50% to 75% of people who use drugs have reported willingness to use and electronic overdose detection system (Ahamad et al., 2019; Kanter et al., 2021) the concern for premature or inadvertent naloxone administration (and subsequent precipitated withdrawal) is expected to be a significant barrier to acceptability of an auto-injector device.

## Opportunities, limitations and future work

The currently available literature suggests that we can use sensors to detect digital biomarkers of opioid ingestion patterns, and the consequences that accompany their use including dependence, withdrawal, hyperalgesia and overdose. But substantial model refinement and expansion are needed to reach clinically acceptable accuracy and to develop interventions that are useful to (and trusted by) clinicians and patients. Digital interventions also need to balance the vast amount of information available with the potential added burden it will create for healthcare providers. Designers need to focus on providing useful and actionable data to appropriate clinicians and other providers at the point in their workflow when they can efficiently use it to impact patient care, i.e. to provide the right data, to the right person at the right time.

Once optimized, these models can be leveraged to develop a clinical tool to monitor opioid use, coupled with behavioral interventions, either with patient facing JITA mobile interventions, clinician facing prescribing recommendations, or both. The resultant digital health system would provide an architectural backbone to deliver both opioid-related pharmacotherapy and behavioral interventions to recognize and react to signs of physiologic adaptations over the entire course of medical opioid therapy. For example, detection of excessive opioid administration after surgery could trigger interactive modules for non-opioid pain management (e.g. meditation, physical therapy exercises or music), while the same signals could be monitored for the onset of opioid dependence. Continuous monitoring of opioid withdrawal symptoms could be used to trigger recommendations for (or even automatic delivery of) buprenorphine or comfort medications titrated to individual needs. And for those with active opioid use, detection of overdose could trigger automated injection of life saving naloxone or a call to emergency medical services.

Several digital health tools are currently available for OUD: common technologies employed include telehealth, mobile delivery of evidence-based interventions (e.g. cognitive behavioral therapy or CBT), social networking and delivery of educational content. The only current Food and Drug Administration (FDA)-approved digital therapeutic for OUD is reSET-O (Pear Theraputics, 2022), which provides mobile CBT and is intended as adjunct to buprenorphine therapy. Telehealth-based platforms such as Workit Health (Workit Health, 2022) provide remote access to providers for therapy and initiation of medication for OUD. Other platforms such as Sober Grid (Sober Grid, 2021) provide coaching, educational content and online support community. To our knowledge, no currently available digital therapeutic leverages physiologic digital biomarkers to trigger or evaluate OUD treatment. Once incorporated, digital biomarkers can make existing technologies more personalized, targeted and precise while decreasing work on the part of the patient. They can serve as triggers for interventions and/or as outcomes to track treatment response.

There are numerous important considerations for real-world applications, a critical one being health equity. In developing generalizable digital biomarker-based tools for OUD, we must consider the factors that contribute to the ever-widening “digital divide”, or the worsening of healthcare inequities driven by technology largely along racial and socioeconomic lines (Saeed & Masters, 2021). From a physical standpoint, accuracy of sensor features, specifically light-based measurements such as photoplethysmography (PPG), can vary based on factors such as skin tone (Bent et al., 2020) highlighting the importance of diversity of participants in training datasets. Real-world implementation of algorithms should account for individual demographics (including sex, race and ethnicity) and treatment characteristics (including pharmacokinetic and pharmacodynamic considerations). The ultimate goal will be personalized models that start with a base model and customize for the individual across the first several events to enhance accuracy.

As socioeconomic factors are just as important as physical features, choice of devices required for a digital diagnostic or therapeutic will also be critical. Models and interventions will likely need to be device agnostic, meaning they can be deployed on low-cost, simple, commercially available devices. The models that drive digital biomarkers are only as good as the data that they are run on, so interruptions in the device data stream or quality provide potential barriers. Wearable sensors are subject to both intentional nonadherence (e.g. removal by intended wearer or placement on another person) and unintentional nonadherence (e.g. lack of data acquisition due to forgetting to wear or charge the device, incorrect/inappropriate wear). Devices need to be aesthetically appealing, easy to use and provide alternative functions to suit the users’ lifestyle to incentivize use (Carreiro et al., 2020; Chapman et al., 2022). Developing device agnostic algorithms (and clinical platforms) will allow wider access and adoption by allowing users to more device choices. Finally, other passive features like the prosody of smartphone use, battery charging status of common digital devices or even geolocation can yield digital phenotypes for various stages of the opioid uses spectrum that can be triangulated with digital biomarkers.

Despite the current knowledge gaps, digital biomarkers of opioid use represent a promising avenue for precision medicine in opioid use. A system driven by empirically derived algorithms could be used to monitor opioid use across the spectrum to support safe prescribing practices, to aid in harm reduction for active opioid users, and finally to support those in recovery from OUD. Once matured, these technologies can be harmonized with existing data sources and be leveraged to ultimately reduce opioid-related deaths.

## Abbreviations

AUC, Area Under the Curve; DSM, Diagnostic and Statistical Manual of Mental Disorders; apps, (mobile) applications; ED, Emergency Department; HER: Electronic health record; HIV, Human Immunodeficiency Virus; JITA, Just-in-time Adaptive Interventions; IV, Intravenous; mHealth, mobile Health; NIH, National Institutes of Health; NIDA, National Institute on Drug Abuse; NMDA, N-methyl-D-aspartate; OIH, Opioid-induced Hyperalgesia; OUD, Opioid Use Disorder; QST, quantitative sensory testing; ROC, Receiver Operator Characteristic; RFID, Radiofrequency identification; US, United States

## Data sharing statement

Data sharing is not applicable to this article as no new data were created or analyzed in this study.
